# A preliminary examination of the bacterial, archaeal, and fungal rhizosphere microbiome in healthy and *Phellinus noxius*‐infected trees

**DOI:** 10.1002/mbo3.1115

**Published:** 2020-09-24

**Authors:** Karen Sze Wing Tsang, Man Kit Cheung, Regent Yau Ching Lam, Hoi Shan Kwan

**Affiliations:** ^1^ School of Life Sciences The Chinese University of Hong Kong Shatin Hong Kong; ^2^ Department of Surgery The Chinese University of Hong Kong Shatin Hong Kong; ^3^ Muni Arborist Limited Lam Tsuen Hong Kong

## Abstract

*Phellinus noxius* is a pathogenic fungus that causes brown root rot disease, resulting in a widespread tree and crop mortality in the tropics and subtropics. Early stages of this disease are largely asymptomatic, hindering early diagnosis and effective treatment. We hypothesized that *P*.* noxius* infection would alter the rhizosphere microbiome of infected trees, based on which diagnostic biomarkers could be developed. Here, we examined for the first time the bacterial, archaeal, and fungal rhizosphere microbiome in four species of healthy and *P*.* noxius*‐infected trees (*Ficus microcarpa*, *Celtis sinensis*, *Mallotus paniculatus*, and *Cinnamomum camphora*) using high‐throughput amplicon sequencing. Results revealed the dominance of *Proteobacteria* and *Actinobacteria* in bacteria, *Crenarchaeota* and *Euryarchaeota* in archaea, and *Ascomycota* and *Basidiomycota* in fungi. *Phellinus noxius* infection did not affect the alpha diversity of the bacterial rhizosphere microbiome in all four tree species but affected that of archaea and fungi in a tree species‐dependent manner. Infection with *P*.* noxius* only affected the bacterial rhizosphere composition in *M*.* paniculatus* but not the other three tree species. By contrast, *P*.* noxius* infection affected the composition of the archaeal and fungal rhizosphere microbiome in all four tree species. Collectively, these results suggest that potential diagnostic biomarkers for brown root rot disease are tree species‐specific and should be developed based on different taxonomic groups. Our study has provided insights into the rhizosphere microbiome in healthy and *P*.* noxius*‐infected trees and laid a solid foundation for future comprehensive studies.

## Graphical abstract

*Phellinus noxius* is a pathogenic fungus that causes the devastating brown root rot disease. We examined the bacterial, archaeal, and fungal rhizosphere microbiome in healthy and *P. noxius*‐infected trees of four species commonly found in Hong Kong using high‐throughput amplicon sequencing. Our results revealed distinct effects of *P. noxius* infection on the diversity and composition of the rhizosphere microbiome across host tree species, suggesting that potential diagnostic biomarkers for brown root rot disease are tree species‐specific.

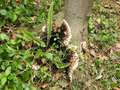

## 1 INTRODUCTION

Brown root rot is a devastating disease prevalent in many tropical and subtropical countries and is caused by the pathogenic white‐rot basidiomycete fungus *Phellinus noxius*. This fungus has a wide host range of over 260 species of trees (Ann, Chang, & Ko, 2002) and crops (Ann, Lee, & Huang, 1999; Ann, Lee, & Tsai, 1999; Farid, Lee, Maziah, & Patahayah, 2009). It causes irrevocable and fatal damage to the plant hosts by targeting their water‐transport system, culminating in root mortality and compromising stability (Hodges & Tenorio, 1984). The life cycle of *P*.* noxius* is similar to other root‐rotting basidiomycetes—a new infection starts from previously infected plants or colonized wood debris, from which the mycelium of *P*.* noxius* grows to infect the lateral and taproots of the host tree (Ann et al., 2002).

*Phellinus noxius* is difficult to eradicate due to its ability to survive on decayed root tissue in the soil for over 10 years (Chang, 1996). Early stages of brown root rot disease are largely asymptomatic, hindering early diagnosis and effective treatment and resulting in a high mortality rate of infected plants. Visible symptoms such as chlorosis and crown dieback can only be seen at the late stages of infections, in which the majority of roots have already been destroyed (Ann et al., 2002; Sahashi, Akisa, Ishihara, Abe, & Morita, 2007). To date, there is no standard curative measure for this disease and most research has been focused on its management, such as the use of biocontrol agents or fumigants (Chang & Chang, 1999; Gohet, Van Canh, Louanchi, & Despreaux, 1991; Prasad & Naik, 2002; Schwarze, Jauss, Spencer, Hallam, & Schubert, 2012). New methods for early diagnosis of the disease are needed.

The rhizosphere microbiome is important to plant health. Microorganisms in the rhizosphere form a symbiotic relationship with the plant hosts, aiding phosphorus and nitrogen uptake, and weathering of minerals, among others (Berendsen, Pieterse, & Bakker, 2012; Trivedi, Van Nostrand, Albrigo, Zhou, & Wang, 2012). Plant exudates can alter the rhizosphere microbiome composition by recruiting specific microorganisms for defense against invasive pathogens (Gu et al., 2016; Pascale, Proietti, Pantelides, & Stringlis, 2020; Wei et al., 2018; Weston et al., 2012; Zhang et al., 2011). These beneficial microbes recruited, for example, *Pseudomonas*, *Bacillus*, and *Trichoderma*, can produce different elicitors and trigger induced systemic resistance (ISR) of the plant hosts via a complex network of defense‐related hormone signaling pathways and thereby making them resistant against pathogenic threats (Pascale et al., 2020). To date, the majority of rhizosphere microbiome studies have focused on healthy plants and mostly on the bacterial communities (e.g. Chaparro, Badri, & Vivanco, 2013; Chapelle, Mendes, Bakker, & Raaijmakers, 2016). Rhizosphere microbiome studies on archaea and fungi are limited. These three kingdoms interact with each other and play important roles in nutrient cycling and soil upkeep, and are therefore important to be studied together (Kirk et al., 2004). Until now, there were no studies of the rhizosphere microbiome concerning *P*.* noxius* infection.

In this study, we examined the bacterial, archaeal, and fungal rhizosphere microbiome in healthy and *P*.* noxius*‐infected trees of four species commonly found in Hong Kong. Our aims were (a) to characterize and compare the diversity and composition of the rhizosphere microbiome in healthy and *P*.* noxius*‐infected trees and (b) to examine whether the changes in the rhizosphere microbiome due to *P*.* noxius* infections are consistent across host tree species. We hypothesized that there would be clear differences in the rhizosphere microbiome between trees with different health status.

## 2 MATERIALS AND METHODS

### 2.1 Soil sampling

Rhizosphere soil samples of *P*.* noxius*‐infected trees belonging to *Ficus microcarpa* (*n* = 3), *Celtis sinensis* (*n* = 1), *Mallotus paniculatus* (*n* = 1), and *Cinnamomum camphora* (*n* = 1) were collected around Hong Kong (22°18′N, 114°12′E) (Table 1). These tree species, especially *F*.* microcarpa*, are commonly found in Hong Kong and are vulnerable to *P*.* noxius* infection. *Phellinus noxius*‐infected trees were initially identified by visual symptoms of chlorosis, crown dieback, the presence of basidiocarps, and the characteristic brown webbing throughout the roots after they were cut open. Rhizosphere samples were collected in triplicate around each tree, 5 cm below the soil surface to avoid surface contamination. Large roots were exposed carefully and soil attached to the roots was sampled with a small shovel. For each sample, a small portion of roots was also taken back to the laboratory for confirmatory tests of *P*.* noxius* infection. Rhizosphere samples were also collected from one healthy tree for each of the four tree species from proximal areas for comparison purposes. In total, 30 rhizosphere soil samples were examined in this study. In the laboratory, ~2 g of soil subsamples from each sample were oven‐dried at 105 °C overnight and then used for total carbon and total nitrogen content analysis in triplicate on the vario MICRO cube elemental analyzer (Elementar, Langenselbold, Hesse, Germany).

**TABLE 1:** Collection locations and physicochemical parameters of rhizosphere samples of healthy and *Phellinus noxius*‐infected trees

Tree species	Health status	Collection location in Hong Kong	Total C (%)	Total N (%)
*Mallotus paniculatus*	Diseased	Sau Nga Road Playground (22°19′19″N, 114°13′37″E)	1.00 ± 0.08	0.07 ± 0.02
*Mallotus paniculatus*	Healthy	Sau Nga Road Playground (22°19′19″N, 114°13′37″E)	1.59 ± 0.54	0.12 ± 0.06
*Celtis sinensis*	Diseased	King's Park (22°18′45″N, 114°10′27″E)	2.59 ± 1.75	0.23 ± 0.16
*Celtis sinensis*	Healthy	King's Park (22°18′45″N, 114°10′27″E)	1.17 ± 0.48	0.07 ± 0.03
*Cinnamomum camphora*	Diseased	The Chinese University of Hong Kong (22°25′10″N, 114°12′24″E)	1.21 ± 0.02	0.09 ± 0.02
*Cinnamomum camphora*	Healthy	The Chinese University of Hong Kong (22°25′10″N, 114°12′24″E)	1.58 ± 0.43	0.11 ± 0.04
*Ficus microcarpa*	Diseased	Kowloon Park (22°17′58″N, 114°10′14″E)	1.54 ± 0.61	0.12 ± 0.05
*Ficus microcarpa*	Diseased	Kowloon Park (22°17′58″N, 114°10′14″E)	1.62 ± 0.52	0.12 ± 0.04
*Ficus microcarpa*	Diseased	Kowloon Park (22°17′58″N, 114°10′14″E)	1.31 ± 0.20	0.08 ± 0.01
*Ficus microcarpa*	Healthy	Kowloon Park (22°17′58″N, 114°10′14″E)	2.51 ± 1.27	0.15 ± 0.07

Note

The values of total carbon and total nitrogen contents are in mean ± standard deviation.

### 2.2 *Phellinus noxius* infection confirmation

Portions of tree roots were washed in distilled water, placed on 2% malt extract agar amended with gallic acid, streptomycin, benomyl, and dichloran (Chang, 1995), and incubated in the dark at 28°C. Pure cultures were then obtained and DNA extracted using DNeasy Plant Mini Kit (Qiagen, Germantown, MD, USA) following the manufacturer's instructions. PCR amplifications were performed using two *P*.* noxius*‐specific primer sets: G1F (5′‐GCC CTT TCC TCC GCT TAT TG‐3′) and G1R (5′‐ CTT GAT GCT GGT GGG TCT CT‐3′) (Wu et al., 2009), and PN‐1F (5′‐AGT TTG CGC TCA TCC ATC TC‐3′) and PN‐2R (5′‐AGCCGACTTACGCCAGCAG‐3′) (Tsai, Hsieh, Ann, & Yang, 2007). Each of the 25 μl PCR mixture consisted of 1× of Green GoTaq Reaction Buffer, 0.8 mM of dNTP, 0.5 μM of primers, 1.25 U of GoTaq DNA Polymerase, and ~0.1 μg of DNA. The PCR regime contained an initial denaturation at 95°C for 2 min, followed by 30 cycles of denaturation at 95°C for 1 min, annealing at 55°C for 30 s and extension at 72°C for 30 s, and a final extension step at 72°C for 5 min. PCR products were then visualized with electrophoresis on 1.5% agarose gels.

### 2.3 DNA extraction, PCR, and amplicon sequencing

Total DNA was extracted from the rhizosphere samples using MoBio PowerSoil DNA Extraction Kit (MO BIO Laboratories, Carlsbad, CA, USA) according to the manufacturer's instructions. The procedure was slightly modified with the additional use of TissueLyser (Qiagen, Germantown, MD, USA) at 30 Hz for 1 min per side to improve cell lysis (Cheung, Wong, Chu, & Kwan, 2018). The V1‐V2 hypervariable region of the bacterial 16S rRNA gene was amplified using primers 28F (5′‐GAG TTT GAT CNT GGC TCA G‐3′) (Handl, Dowd, Garcia‐Mazcorro, Steiner, & Suchodolski, 2011) and 338R (5′‐GCT GCC TCC CGT AGG AGT‐3′) (Amann, Ludwig, & Schleifer, 1995). For archaea, the V1‐V2 region of the 16S rRNA gene was amplified using primers 21F (5′‐TTC CGG TTG ATC CYG CCG GA‐3′) (DeLong, 1992) and Pro341R (5′‐CTG STG CVN CCC GTA GG‐3′) (Takahashi et al., 2014). For fungi, the internal transcribed spacer 1 (ITS1) region was amplified using primers ITS1F (5′‐CTT GGT CAT TTA GAG GAA GTA A‐3′) (Gardes & Bruns, 1993) and ITS2R (5′‐ GCT GCG TTC TTC ATC GAT GC‐3′) (White, Bruns, Lee, & Taylor, 1990). Each PCRmixture consisted of 1× Phusion HF Buffer, 0.5 μM of primers, 200 μM of dNTPs, 1 U of Phusion High‐Fidelity DNA polymerase (New England Biolabs, Ipswich, MA, USA), and 50 ng of DNA. Bacterial 16S rRNA amplifications were performed with an initial denaturation of 98°C for 3 min, followed by 33 cycles of denaturation at 98°C for 10 s, annealing at 61°C for 30 s and extension at 72°C for 30 s, and a final extension step at 72°C for 10 min. For archaea, an annealing temperature of 67°C was used, and for fungi, an annealing temperature of 55°C with 35 cycles was used. PCR products were quality‐checked on 1.5% agarose gels, purified with QIAquick Gel Extraction Kit (Qiagen, Germantown, MD, USA) and sequenced from the forward primer end on an Ion Torrent PGM (318 chip v2) at the Core Facilities of The Chinese University of Hong Kong.

### 2.4 Sequence analysis

Raw sequence reads were demultiplexed, filtered for quality, and analyzed using QIIME 1.9.1 (Caporaso et al., 2010) as previously described (Cheung et al., 2015). Chimeric sequences were identified and removed using USEARCH 6.1 (Edgar, 2010) against the “Gold” reference dataset for bacteria and archaea, and against the UNITE dynamic ITS1 reference dataset (2016–01–01 release) (Kõljalg et al., 2013) for fungi. Sequence reads from the same kingdom were clustered into operational taxonomic units (OTUs) at 97% similarity using uclust with the open‐reference OTU picking method. Representative OTUs were aligned to the Greengenes reference dataset (13_8 release) (DeSantis et al., 2006) for bacteria and archaea, and the UNITE dynamic reference dataset (2016–11–20 release) for fungi, and taxonomically assigned using the RDP naïve Bayesian Classifier (Wang, Garrity, Tiedje, & Cole, 2007). Sequence reads of plant origin were removed from further analysis.

### 2.5 Statistical analysis

Before diversity analyses, the bacterial, archaeal, and fungal sequence datasets were rarefied to 13,055, 13,821, and 16,386 reads, respectively. Alpha diversity was estimated with the Shannon index and the number of observed OTUs. Beta diversity was estimated using principal coordinate analysis (PCoA) with the unweighted UniFrac distance for bacteria and archaea and the Bray–Curtis dissimilarity for fungi. All the above statistical tests were conducted using scripts in QIIME. Linear regression analysis was performed using GraphPad Prism 8 (GraphPad Software Inc., San Diego, CA). Differences were considered to be statistically significant when *p* < 0.05.

## 3 RESULTS

### 3.1 Physicochemical parameters

All rhizosphere samples shared a similar total carbon and total nitrogen contents among tree species and between healthy and diseased samples of the same species (Table 1).

### 3.2 Alpha diversity

After quality filtering, a total of 710,349 bacterial 16S rRNA reads (13,055–32,347 per sample), 1,327,491 archaeal 16S rRNA reads (13,821–109,396 per sample), and 1,343,825 fungal ITS reads (16,386–101,826 per sample) were obtained for the 30 rhizosphere soil samples. The Shannon diversity and the number of observed OTUs calculated ranged, respectively, from 11.28–11.85 and 5858–6959 for bacteria, 4.72–6.16, and 384–608 for archaea, and 3.49–5.34 and 550–844 for fungi (Table 2). There was no obvious difference in the Shannon diversity of bacteria between healthy and *P*.* noxius*‐infected trees for all tree species. The archaeal Shannon diversity was also similar between healthy and diseased samples of *C*.* sinensis* and *F*.* microcarpa* but was higher in diseased samples of *M*.* paniculatus* and *C*.* camphora*. The fungal Shannon diversity was lower in diseased samples of all tree species apart from *F*.* microcarpa*.

**TABLE 2:** Alpha diversity of the bacterial, archaeal, and fungal rhizosphere microbiome in healthy and *Phellinus noxius*‐infected trees

Tree species	Health status	Bacteria		Archaea		Fungi	
		Shannon	Observed OTUs	Shannon	Observed OTUs	Shannon	Observed OTUs
*Mallotus paniculatus*	Healthy	11.28 ± 0.23	5857.56 ± 49.65	5.44 ± 0.48	517.63 ± 44.81	5.14 ± 0.91	646.40 ± 91.20
*Mallotus paniculatus*	Diseased	11.74 ± 0.12	6367.70 ± 309.14	6.16 ± 0.51	440.67 ± 71.72	3.83 ± 0.84	616.23 ± 77.70
*Celtis sinensis*	Healthy	11.85 ± 0.03	6864.50 ± 104.34	5.23 ± 0.59	607.97 ± 182.90	5.34 ± 0.23	756.47 ± 98.02
*Celtis sinensis*	Diseased	11.60 ± 0.02	6439.00 ± 54.64	4.86 ± 0.15	442.67 ± 73.38	3.79 ± 0.59	695.27 ± 52.40
*Ficus microcarpa*	Healthy	11.83 ± 0.06	6862.00 ± 112.48	4.99 ± 0.89	383.50 ± 29.50	3.55 ± 0.63	621.46 ± 92.84
*Ficus microcarpa*	Diseased	11.77 ± 0.06	6857.81 ± 304.80	4.72 ± 0.10	445.33 ± 164.08	3.49 ± 0.58	549.50 ± 108.00
*Cinnamomum camphora*	Healthy	11.83 ± 0.05	6958.77 ± 99.03	4.82 ± 0.14	446.33 ± 5.25	5.24 ± 0.17	844.30 ± 1.10
*Cinnamomum camphora*	Diseased	11.70 ± 0.05	6651.20 ± 69.00	6.16 ± 0.35	573.63 ± 165.30	4.79 ± 0.13	738.30 ± 32.72

Note

Values are in mean ± standard deviation.

### 3.3 Beta diversity

**FIGURE 1:**
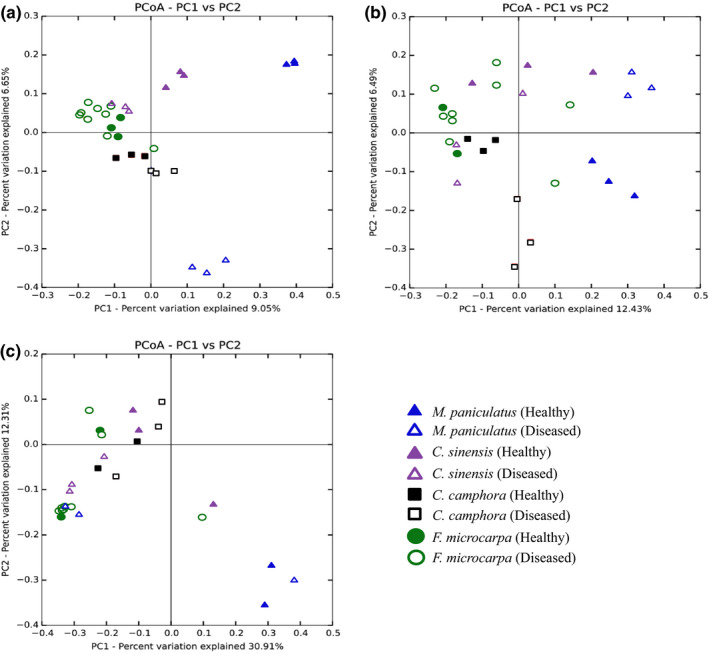
Principal coordinate analysis plots of the bacterial (a), archaeal (b), and fungal (c) rhizosphere microbiome

PCoA revealed that the rhizosphere microbiome structure of samples collected around the same tree were in general similar to each other (Figure 1). For bacteria, samples from the same tree species clustered together, except for *M*.* paniculatus* (Figure 1a). Samples from healthy and *P*.* noxius*‐infected trees also formed distinct clusters for all tree species apart from *F*.* microcarpa*. For archaea, PCoA revealed distinct clusters for *M*.* paniculatus* and *C*.* camphora*, and between healthy and diseased samples of *M*.* paniculatus*, *C*.* sinensis*, and *C*.* camphora* (Figure 1b). For fungi, no clear clusters were formed according to tree species, and healthy and diseased samples were only separated in *C*.* sinensis* (Figure 1c).

### 3.4 Taxonomic composition in healthy trees

**FIGURE 2:**
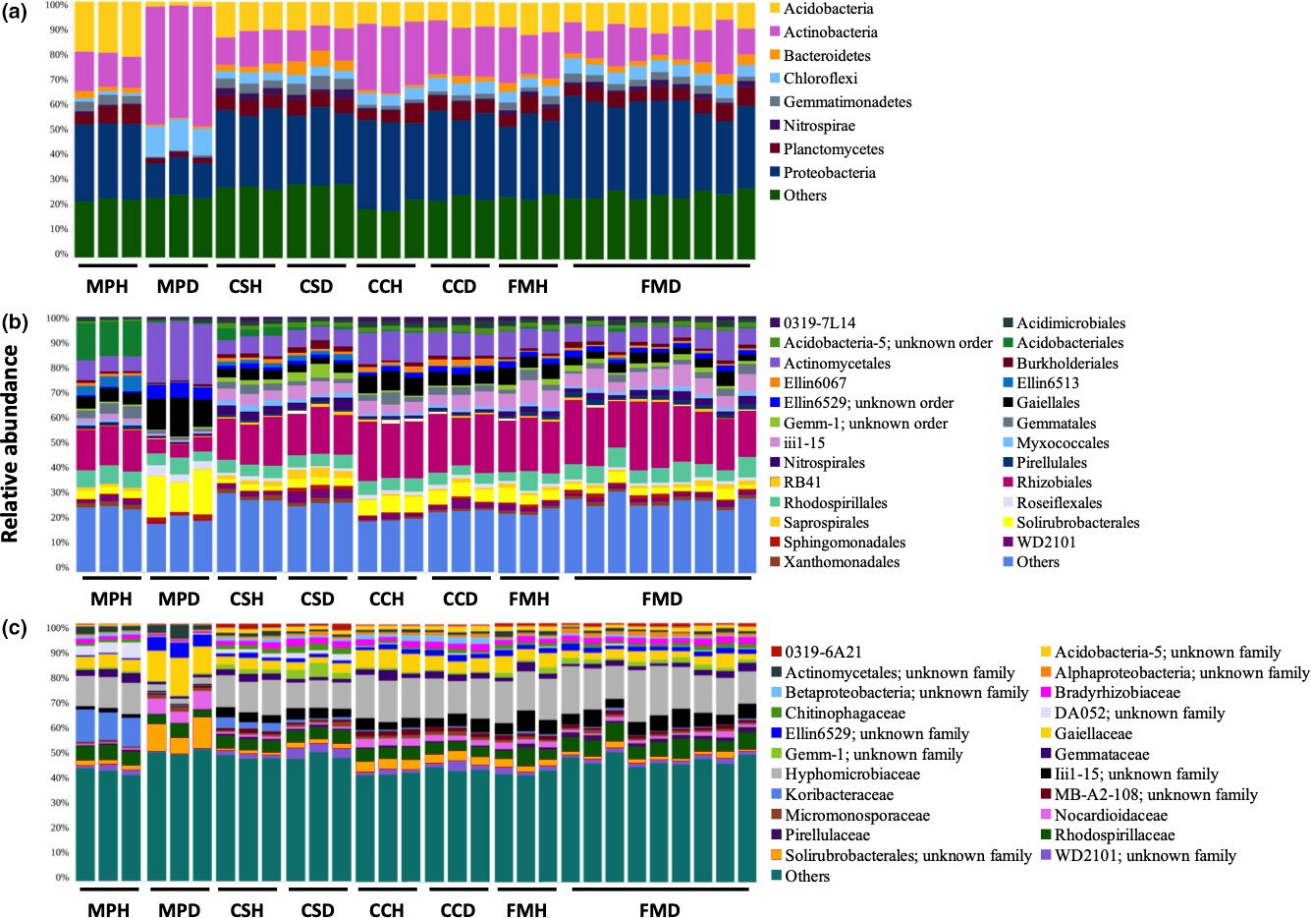
The relative abundance of dominant (>1%) bacterial phyla (a), orders (b), and families (c) in the rhizosphere microbiome of four species of trees. MP: *Mallotus paniculatus*, CS: *Celtis sinensis*, CC: *Cinnamomum camphora*, FM: *Ficus microcarpa*; H: healthy, D: diseased

For bacteria, the eight major (>1% average relative abundance) phyla recovered were *Proteobacteria* (36%), *Actinobacteria* (22%), *Acidobacteria* (12%), *Planctomycetes* (6%), *Chloroflexi* (6%), *Bacteroidetes* (3%), *Gemmatimonadetes* (3%), and *Nitrospirae* (2%), which together accounted for ~90% of all bacterial 16S rRNA reads (Figure 2a). All four tree species were dominated by *Proteobacteria*, *Acidobacteria*, and *Actinobacteria*. At the order level, all four tree species were dominated by *Rhizobiales*, *Rhodospirillales*, *Actinomycetales*, and *Gaiellales* (Figure 2b). Besides, there was an abundant amount of *Acidobacteriales* and *Ellin6513* in *M*.* paniculatus*; *iii1*‐*15* in *C*.* sinensis*; and *iii1*‐*15* and *Solirubrobacterales* in *C*.* camphora* and *F*.* microcarpa*. At the family level, all four tree species were dominated by *Hyphomicrobiaceae*, *Gaiellaceae*, and *Rhodospirillaceae* (Figure 2c). Besides, there was an abundant amount of *Koribacteraceae* in *M*.* paniculatus*.

**FIGURE 3:**
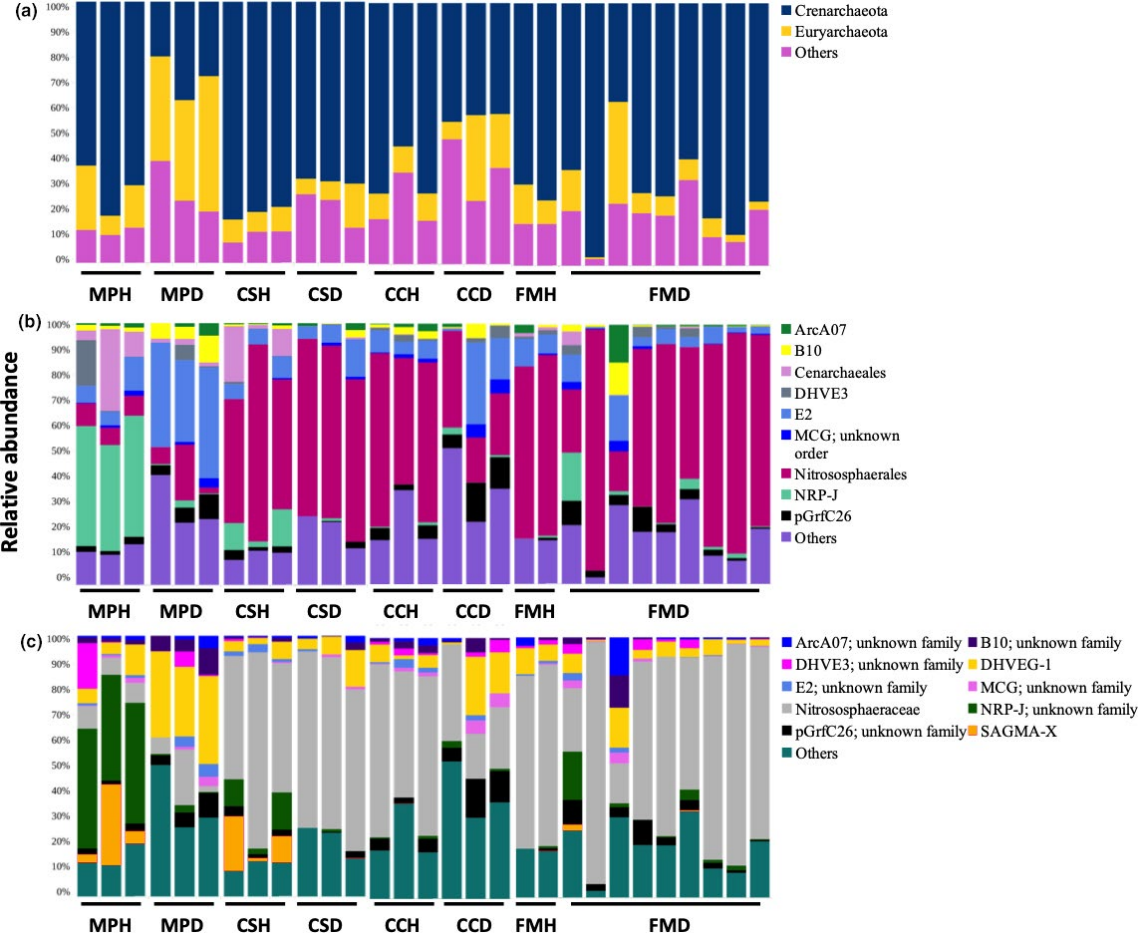
The relative abundance of dominant (>1%) archaeal phyla (a), orders (b), and families (c) in the rhizosphere microbiome of four species of trees. MP: *Mallotus paniculatus*, CS: *Celtis sinensis*, CC: *Cinnamomum camphora*, FM: *Ficus microcarpa*; H: healthy, D: diseased

For archaea, all four tree species were dominated by *Crenarchaeota* and *Euryarchaeota* at the phylum level (Figure 3a). At the order level, *M*.* paniculatus* was dominated by *NRP*‐*J*, whereas *C*.* sinensis*, *C*.* camphora*, and *F*.* microcarpa* were dominated by *Nitrososphaerales* (Figure 3b). Besides, *Cenarchaeales* was dominant in *M*.* paniculatus* and *C*.* sinensis*. At the family level, all tree species apart from *M*.* paniculatus* were dominated by *Nitrososphaeraceae* (Figure 3c). Besides, *SAGMA*‐*X* was dominant in *M*.* paniculatus* and *C*.* sinensis*.

**FIGURE 4:**
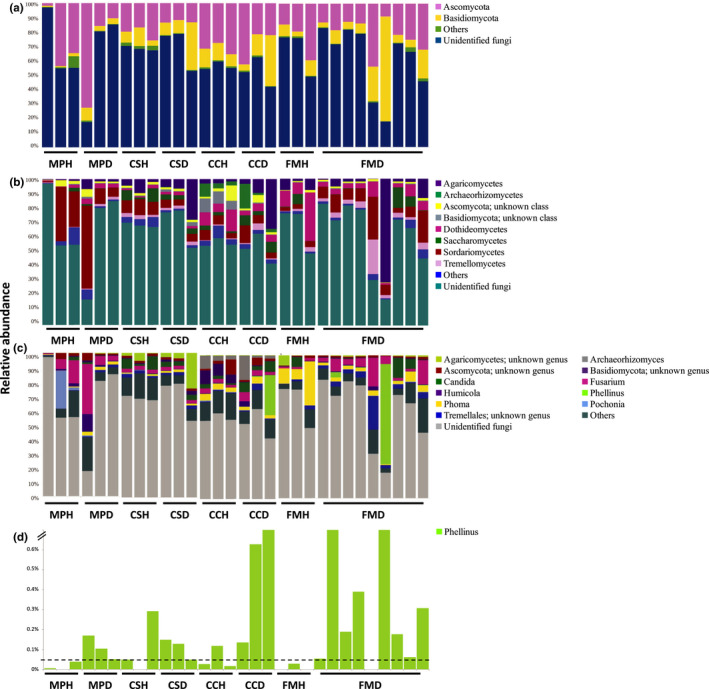
The relative abundance of dominant (>1%) fungal phyla (a), classes (b), and genera (c) as well as the *Phellinus* genus (d) in the rhizosphere microbiome of four species of trees. MP: *Mallotus paniculatus*, CS: *Celtis sinensis*, CC: *Cinnamomum camphora*, FM: *Ficus microcarpa*; H: healthy, D: diseased. The horizontal dotted line in panel (d) denotes the relative abundance at 0.05%

Over 65% of the fungal ITS reads could not be identified to a known fungal phylum. *Ascomycota* and *Basidiomycota* were the most abundant fungal phyla for all four tree species (Figure 4a). At the class level, *M*.* paniculatus* was dominated by *Sordariomycetes*, whereas *C*.* camphora* and *F*.* microcarpa* were dominated by *Dothideomycetes* (Figure 4b). Besides, *C*.* sinensis* and *F*.* microcarpa* contained an abundant amount of *Agaricomycetes*. At the genus level, *M*.* paniculatus* was dominated by *Fusarium* and *Pochonia*, whereas *C*.* sinensis*, *C*.* camphora*, and *F*.* microcarpa* were dominated by *Candida*, *Archaeorhizomyces*, and *Phoma*, respectively (Figure 4c).

### 3.5 Taxonomic composition in *Phellinus noxius*‐infected trees

There was no obvious difference in the taxonomic composition of major bacterial phyla, orders, or families between healthy and *P*.* noxius*‐infected trees of *C*.* sinensis*, *C*.* camphora*, and *F*.* microcarpa* (Figure 2). By contrast, in *P*.* noxius*‐infected trees of *M*.* paniculatus*, there was more *Actinobacteria* and *Chloroflexi* and fewer *Acidobacteria*, *Proteobacteria*, and *Planctomycetes* at the phylum level (Figure 2a); more *Actinomycetales*, *Gaiellales*, and *Solirubrobacterales* and fewer *Acidobacteriales*, *Ellin6513*, *Gemmatales*, and *Rhizobiales* at the order level (Figure 2b); and more *Gaiellaceae* and *Nocardioidaceae* and fewer *Gemmataceae* and *Hyphomicrobiaceae* at the family level (Figure 2c).

For archaea, there was no obvious difference in the taxonomic composition of major phyla between healthy and *P*.* noxius*‐infected trees of *C*.* sinensis* and *F*.* microcarpa* (Figure 3a). By contrast, there was more *Euryarchaeota* and fewer *Crenarchaeota* in *P*.* noxius*‐infected trees of *M*.* paniculatus* and *C*.* camphora*. At the order level, there was more *E2*, *B10*, and *pGrfC26*, and fewer *Cenarchaeales*, *NPR*‐*J* in diseased trees of *M*.* paniculatus*; fewer *Cenarchaeales* and *NRP*‐*J* in diseased trees of *C*.* sinensis*; more *pGrfC26* and fewer *Nitrososphaerales* in diseased trees of *C*.* camphora*; and more *pGrfC26* in diseased trees of *F*.* microcarpa* (Figure 3b). At the family level, there was more *DHVEG*‐*1* and fewer *SAGMA*‐*X* in diseased trees of *M*.* paniculatus*; fewer *SAGMA*‐*X* in diseased trees of *C*.* sinensis*; and more *DHVEG*‐*1* and fewer *Nitrososphaeraceae* in diseased trees of *C*.* camphora* (Figure 3c).

**FIGURE A1:**
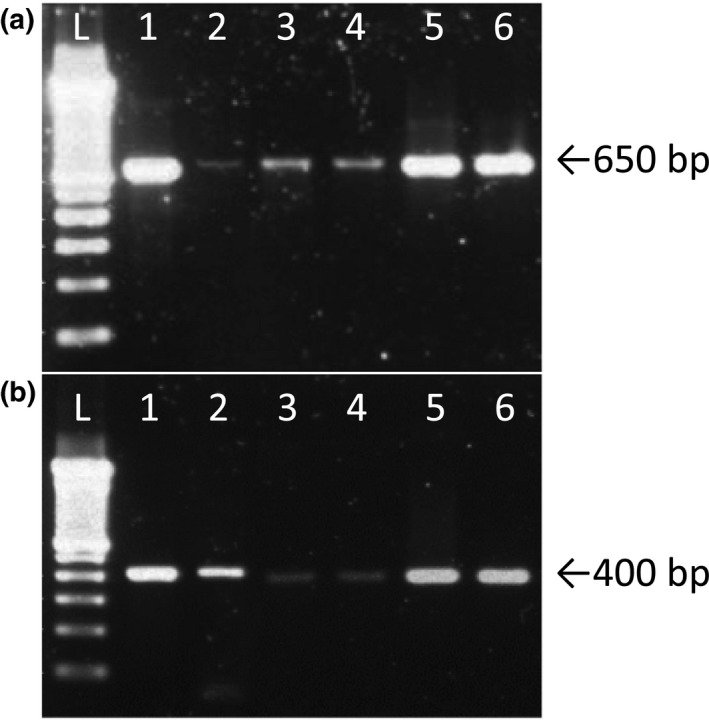
Gel photographs of PCR products amplified using *Phellinus noxius*‐specific primer sets G1F/G1R (a) and PN‐1F/PN‐2R (b) on pure cultures obtained from the roots of *Phellinus noxius*‐infected trees. L: ladder, 1 to 3: *Ficus microcarpa*, 4: *Mallotus paniculatus*, 5: *Cinnamomum camphora*, and 6: *Celtis sinensis*. The expected amplicon size is indicated with arrows

For fungi, there was more *Basidiomycota* in diseased trees of *M*.* paniculatus* and fewer *Ascomycota* in diseased trees of *C*.* sinensis* (Figure 4a). At the class level, there was more *Agaricomycetes* and *Dothideomycetes* in diseased trees of *M*.* paniculatus*; more *Agaricomycetes* and fewer *Sordariomycetes* in diseased trees of *C*.* sinensis*; more *Agaricomycetes* and *Saccharomycetes* and fewer *Dothideomycetes* in diseased trees of *C*.* camphora*; and more *Sordariomycetes* and *Tremellomycetes* and fewer *Dothideomycetes* in diseased trees of *F*.* microcarpa* (Figure 4b). At the genus level, there was more *Phoma* and fewer *Pochonia* in diseased trees of *M*.* paniculatus*, and more *Fusarium* and fewer *Phoma* in diseased trees of *F*.* microcarpa* (Figure 4c). Moreover, sequences from the *Phellinus* genus were detected in all except one *P*.* noxius*‐infected rhizosphere samples, with a relative abundance ranging from 0.05% to 70.1% (Figure 4c,d). By contrast, most healthy samples contained <0.05% of *Phellinus* reads. Nonetheless, it should be reminded that all trees in this study were selected according to visible symptoms, with the health status of diseased trees further confirmed with *P*.* noxius*‐specific PCRs (Figure A1).

**FIGURE A2:**
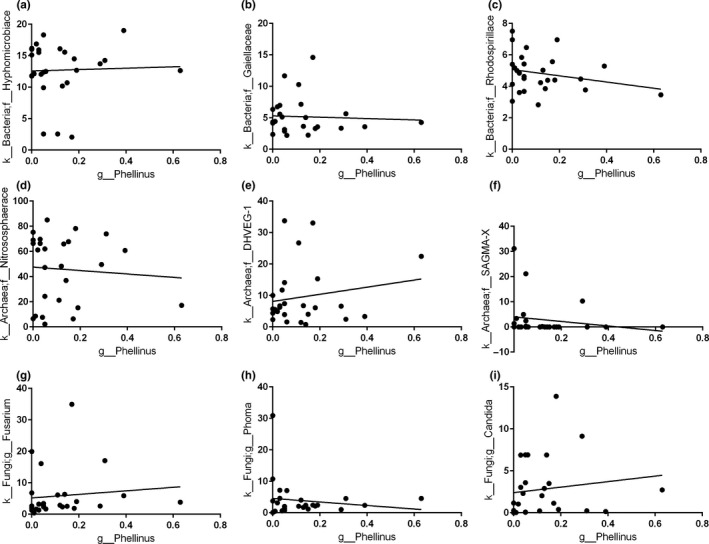
Correlation between the relative abundance of the *Phellinus* genus and the bacterial families *Hyphomicrobiaceae* (a), *Gaiellaceae* (b), and *Rhodospirillaceae* (c), the archaeal families *Nitrososphaeraceae* (d), *DHVEG*‐*1* (e), and *SAGMA*‐*X* (f), and the fungal genera *Fusarium* (g), *Phoma* (h), and *Candida* (i). Samples with a relative abundance of *Phellinus* >3% were regarded as outliers and excluded from the analysis

Linear regression analysis revealed no significant correlation between the relative abundance of the *Phellinus* genus and the dominant bacterial families *Hyphomicrobiaceae*, *Gaiellaceae*, and *Rhodospirillaceae*, the dominant archaeal families *Nitrososphaeraceae*, *DHVEG*‐*1*, and *SAGMA*‐*X*, or the dominant fungal genera *Fusarium*, *Phoma*, and *Candida* (*p* > 0.05, *F* test) (Figure A2).

## 4 DISCUSSION

In this study, we have examined for the first time the effects of *P*.* noxius* on the structure and diversity of the bacterial, archaeal, and fungal rhizosphere microbiome in trees. Until now, only a few rhizosphere microbiome studies have simultaneously investigated these three taxonomic groups, and the majority of them have studied only the healthy rhizosphere of trees (Uroz et al., 2016; Veach et al., 2019). By contrast, studies involving diseased samples usually focus on agricultural crop plants (Filion, Hamelin, Bernier, & St‐Arnaud, 2004; Han et al., 2017; Li, Ren, Jia, & Dong, 2014; Wei et al., 2018; Zhang et al., 2011), but none of them has examined the archaeal communities. Information on the rhizosphere microbiome across multiple kingdoms is essential due to their integral roles in nutrient cycling and the ability to maintain symbiotic and antagonistic relationships with the plant hosts. Our results have demonstrated that *P*.* noxius* can alter the rhizosphere microbiome of healthy trees but the effects depend on the species of trees. However, a major limitation of this study is the lack of true biological replicates for each tree species, which has hindered statistical testing on the differences observed.

All rhizosphere samples examined in this study were abundant in *Proteobacteria*. This is not unexpected as members of this bacterial phylum are fast‐growing (Fierer, Bradford, & Jackson, 2007). Similar results have also been reported in other soil microbiome studies of trees (Uroz et al., 2016) and crops (Fu et al., 2017; Li et al., 2014). Comparing healthy and diseased samples in all four tree species together, no clear clustering between samples of healthy and *P*.* noxius*‐infected trees was observed. This suggests that the effect of host tree species on the rhizosphere composition is larger than that resulting from *P*.* noxius* infection. Comparing healthy and diseased samples in each tree species separately, only *M*.* paniculatus* showed compositional changes in the bacterial microbiome; *M*.* paniculatus* and *C*.* camphora* showed changes in the archaeal microbiome, and *C*.* camphora* and *F*.* microcarpa* showed changes in the fungal microbiome. The profiles of all three kingdoms were not different between healthy and diseased samples of *C*.* sinensis*. This suggests that the effects of *P*.* noxius* infection on the rhizosphere microbiome vary across kingdoms and depend on the host tree species. Notably, members from the phylum *Chloroflexi* were more abundant in diseased samples of *M*.* paniculatus* (Figure 2a). This could represent a direct response to the invasion as *Chloroflexi* members are often found in disease suppressive soils and have been suggested to be part of the host plant defense system due to their ability to prevent iron uptake and root colonization by fungal plant pathogens (Lemanceau & Alabouvette, 1993; Liu et al., 2016; Rodriguez & Fraga, 1999). However, it is also possible that some *Chloroflexi* members are opportunistic pathogens that were enriched in the diseased samples. The archaeal order *E2* was enriched in the diseased samples of *M*.* paniculatus* (Figure 3b). *E2* comprises uncultured members of potential methanogens and its enrichment in the diseased samples might be related to an increase of detritus and carbon sources resulting from root decay (Gannes, Eudoxie, Bekele, & Hickey, 2015; Iino et al., 2013). For fungi, the genus *Fusarium* was enriched in the diseased samples of *F*.* microcarpa* (Figure 4c). *Fusarium* comprises plant growth‐promoting members that can trigger ISR for enhancing the defense of the plant host upon pathogen attack (Pascale et al., 2020).

Infection with *P*.* noxius* did not affect the bacterial diversity in the rhizosphere of all four tree species examined here. This differs from other studies on diseased microbiomes in crops (Han et al., 2017; Li et al., 2014; Shang et al., 2016; Wei et al., 2018), tree seedlings (Filion et al., 2004), and shrubs (Zhang et al., 2011). For example, wilted Lanzhou Lily has a higher rhizosphere bacterial diversity (Shang et al., 2016), whereas diseased black spruce (*Picea mariana*) seedlings (Filion et al., 2004) and cotton plants (Zhang et al., 2011) have a lower rhizosphere bacterial diversity. This suggests that, in contrast to the general belief that microbial diversity can act as a biomarker for plant health (Berg et al., 2017), for *P*.* noxius* infection, the rhizosphere bacterial diversity is not a reliable indicator of plant health. Moreover, the fact that the effects of *P*.* noxius* infection on the archaeal and fungal diversity are different across host tree species suggests that the diversity of different microbial taxonomic groups should be examined for different host tree species to indicate the status of *P*.* noxius* infection.

Differences in the microbiome composition between healthy and diseased rhizospheres have been reported in some previous studies of crop plants (Li et al., 2014; Shang et al., 2016; Wei et al., 2018). However, no obvious differences are observed for most tree species in this study. The differences in responses could be explained by species‐dependent microbial recruitment (Turner et al., 2013) or disparities in the stages of infection (Zhang et al., 2011). A previous study on apple replant disease also shows no changes in the bacterial and fungal rhizosphere microbiome in diseased apple trees (Jiang et al., 2017). A possible reason for the lack of changes in some of our samples is the physical difference between trees examined in this study and plants in previous ones. Compared to smaller crop plants that have a simple root system, all trees examined here are over seven meters tall with an extensive root system, comprising both fresh and decaying roots even when the infection is serious. Among the four tree species examined here, *M*.* paniculatus* showed the most significant changes in its bacterial and archaeal rhizosphere communities. The trunk diameter, height, and root radius of *M*.* paniculatus* were the smallest among all four tree species examined in this study. The smallest and simplest root system of *M*.* paniculatus* might explain why *P*.* noxius* infection affected its rhizosphere microbiome more than the other three tree species. In particular, all *F*.* microcarpa* trees examined in this study are with a diameter at breast height (DBH) of >1000 mm (Leisure & Cultural Services Department, 2016). *Ficus microcarpa* is known for its large root network and abundance of aerial roots, which constantly produce fresh roots (Zimmerman, Wardrop, & Tomlinson, 1968). Therefore, *P*.* noxius* infection may exert a smaller effect on its rhizosphere microbiome as roots unaffected by the infection may produce exudates that diffuse to the nearby rhizosphere of the affected roots. Nonetheless, further studies are needed to understand more clearly how the tree rhizosphere microbiome changes when a pathogen invades.

In conclusion, we have examined here the effects of *P*.* noxius* on the diversity and composition of the bacterial, archaeal, and fungal rhizosphere microbiomes in four tree species. We showed that *P*.* noxius* infection did not affect the alpha diversity of the bacterial rhizosphere microbiome in all four tree species but affected that of archaea and fungi in a tree species‐dependent manner. The effects of *P*.* noxius* infection on the composition of the three kingdoms also varied with host tree species. Our study has provided insights into the rhizosphere microbiome in healthy and *P*.* noxius*‐infected trees and showed that potential diagnostic biomarkers for brown root rot disease are likely tree species‐specific. Future studies employing more host tree species and a larger sample size per species should revisit these preliminary findings with sophisticated statistical tests. With this, we anticipate the development of rhizosphere microbiome‐based diagnostic biomarkers to facilitate early detection of the devastating brown root rot disease in the near future.

## CONFLICT OF INTERESTS

None declared.

## AUTHOR CONTRIBUTIONS

**Karen Sze Wing Tsang:** Formal analysis (lead); funding acquisition (lead); investigation (lead); methodology (lead); visualization (equal); writing – original draft (equal). **Man Kit Cheung:** Funding acquisition (supporting); visualization (equal); writing – original draft (equal); writing – review & editing (equal). **Regent Yau Ching Lam:** Investigation (supporting); resources (supporting). **Hoi Shan Kwan:** Conceptualization (lead); funding acquisition (supporting); methodology (supporting); resources (lead); supervision (lead); writing – review & editing (equal).

## ETHICS STATEMENT

None required.

## Data Availability

Raw sequence reads generated in this study are available in the NCBI Sequence Read Archive (SRA) under the accession PRJNA433610. https://www.ncbi.nlm.nih.gov/biopr​oject/​PRJNA​433610.
